# Postpartum practices of puerperal women and their influencing factors in three regions of Hubei, China

**DOI:** 10.1186/1471-2458-6-274

**Published:** 2006-11-07

**Authors:** Nian Liu, Limei Mao, Xiufa Sun, Liegang Liu, Banghua Chen, Qiang Ding

**Affiliations:** 1 Department of Nutrition and Food Hygiene, School of Public Health, Tongji Medical College, Huazhong University of Science & Technology, 13 Hangkong Road, Wuhan 430030, P. R. China

## Abstract

**Background:**

'Sitting month' is a Chinese tradition for women's postpartum custom. The present study aims to explore the postpartum dietary and health practices of puerperal women and identify their influential factors in three selected regions of Hubei, China.

**Methods:**

A cross-sectional retrospective study was conducted in the selected urban, suburban and rural areas in the province of Hubei from 1 March to 30 May 2003. A total of 2100 women who had given birth to full-term singleton infants in the past two years were selected as the participants. Data regarding postpartum practices and potentially related factors were collected through questionnaire by trained investigators.

**Results:**

During the puerperium, 18% of the participants never ate vegetables, 78.8% never ate fruit and 75.7% never drank milk. Behaviour taboos such as no bathing, no hair washing or teeth brushing were still popular among the participants. About half of the women didn't get out of the bed two days after giving birth. The average time they stayed in bed during this period was 18.0 h. One third of them didn't have any outdoor activities in that time periods. The educational background of both women and their spouses, location of their residence, family income, postnatal visit, nutrition and health care educational courses were found to be the influencing factors of women's postpartum practices.

**Conclusion:**

Traditional postpartum dietary and health behaviours were still popular among women in Hubei. Identifying the factors associated with traditional postpartum practices is critical to develop better targeting health education programs. Updated Information regarding postpartum dietary and health practices should be disseminated to women.

## Background

The postpartum period, or puerperium, starts about an hour after the delivery of the placenta and includes the following six weeks [1]. By six weeks after delivery, most of the changes of pregnancy, labor, and delivery have resolved and the body has reverted to the nonpregnant state [1,2]. The postpartum period is a very special phase in the life of a woman. Her body needs to heal and recover from pregnancy and childbirth. A good postpartum care and well balanced diet during puerperal period is very important for the health of a woman.

According to Chinese traditions, the first 30 or 40 days postpartum is recognized as a special time period for behaviour restrictions and a state for convalescence. This period is called 'sitting month' or 'doing the month'. Based on Chinese traditional medicine, postpartum women are in a 'weak' state because of 'Qi' deficiency and blood loss [3]. Their body can be easily attacked by 'heat' or 'cold ', which may cause some health problems like dizziness, headache, backache and arthragia in the month or in later years. Therefore, Chinese women are advised to follow a specific set of food choices and health care practices. For example, the puerperal women should stay inside and not go outdoors; all windows in the room should be sealed well to avoid wind. Bathing and hair washing should be restricted to prevent possible headache and body pain in later years. Foods such as fruits, vegetables, soybean products and cold drinks which are considered 'cold' should be avoided [4,5]. In contrast, foods such as brown sugar, fish, chicken and pig's trotter which are considered 'hot' should be encouraged [5]. It is believed that if a woman does not observe these restrictions, she may suffer a poor health at her later life. These traditional postnatal believes and practices are often passed down from senior females in the family to the younger generations [4].

The postpartum dietary and lifestyle habits vary greatly among different countries and cultures [4,7-9]. In western countries, instead of restrictions, women are encouraged to eat a well-balanced diet from all food categories and start physical exercises during this period [1,6]. In the past few decades, China experiences fast economic growth and cultural changes as well. The traditional postpartum practices face the challenges from western culture. Despite a few studies done on the postpartum practices in north China [10-13], there was no such one in Hubei. Furthermore, none of the previous study had tried to identify the influential factors of puerperal practices. The purpose of this research aimed to study the prevalence of postpartum practices and identify correlated biological and social factors during the puerperium in Hubei, China. We believe that this study will provide insightful information for health care policy makers, social workers and health care providers to develop better strategies on health education programs.

## Methods

### Study sites and participants

A cross- sectional retrospective survey of postpartum practices during the puerperium was conducted in the central part of China from 1 March to 30 May 2003. The study was carried out in Hubei, a province with 60 million residents and the economic development was at middle level among all provinces in China. People's life styles varied considerably between metropolitan and rural areas. The rural areas remain underdeveloped economically and reserved culturally. Thus three regions representing urban, suburban and rural areas, respectively, have been selected. A total of 2100 women who had given birth to full-term singleton infants in the previous two years were chosen as the participants by cluster-stratified sampling. The study protocol was approved by the local health Department and the Institutional Review Board of Tongji Medical College, China. The study was fully explained to the participants before they could give their consent to participate.

### Data collection

After obtaining informed consent, the interviewer administered a pre-tested questionnaire that collected historical data on social-demographic characters, obstetric history, physical activity, dietary and health behaviours during the puerperium. A retrospective food frequency questionnaire was also performed to collect information on dietary intake during this period. The questionnaire consisted of 16 food categories. The participants were asked to recall the frequency and approximately the amount of food consumed over the puerperal period. Food quantities were estimated in grams and milliliters. Cups, bowls and spoons were used to help recalling and measuring foods. The frequency of consuming a particular type of food was recorded on a daily, weekly or monthly basis (times/day, times/week, times/month). The interviewers were health professionals from local maternal and child health clinics, who were intensively trained by our research team.

### Statistical analysis

Using the food frequency data, the overall amount of a particular type of food consumed during the puerperium was calculated and converted as mean daily intake. All statistical analyses were performed using SAS 8.1 statistical software package. Frequency distributions were used to describe characteristics of women with respect to demographics, dietary and health behaviours. A series of multiple logistic regression analyses were performed to estimate the effects of various influencing factors on specific dietary or health behaviours. In the multivariable logistic regression model, all potential influencing factors were initially included into the models. Stepwise regression procedures were used to determine which factors were most strongly correlated with the outcomes of interests. Only the variables that had a significant relationship with the response variables at the *P *< 0.05 level were then entered as independent variables to be included in each final regression model. The criterion for removal in the regression analysis was *P *> 0.1. The following variables were considered potential independent variables: age (continuous), parity (0 = primipara, 1 = multipara), woman's education level (1 = primary school and lower, 2 = middle school, 3 = high school, 4 = college or higher), husband's education level (1 = primary school and lower, 2 = middle school, 3 = high school, 4 = college or higher), urban (0 = no, 1 = yes), suburban (0 = no, 1 = yes), rural (0 = no, 1 = yes), annual family income per capita (continuous), attending nutrition education courses (0 = no, 1 = yes), belief that vegetable and fruit are cold nature (0 = no, 1 = yes), delivery ways (0 = vaginal delivery, 1 = cesarean delivery), serious obstetric complications (0 = no, 1 = yes), dietary arrangement (0 = somebody else, 1 = mother or mother-in-law), prenatal examination (0 = no, 1 = yes), postnatal visit (0 = no, 1 = yes), attending health care education courses (0 = no, 1 = yes).

## Results

### Characteristics of the participants

Of 2100 women recruited, a total of 1975 completed the questionnaire (638 in the urban, 627 in the suburban and 710 in the rural area), with response rate of 94.1%. The age range of the samples was from 18 to 44 years, with a mean age of 26.8 ± 3.6 years. The annual family income per capita was 5178.8 Yuan in the city, 2623.2 Yuan in the suburb and 1798.5 Yuan in the rural. Most of the women were primiparous. Other characteristics of the participants are shown in Table 1.

### Dietary behaviours

#### Prevalence of dietary behaviours

During the puerperium, foods that women most frequently consumed were: egg, brown sugar, carassius fish, poultry, pig's trotter and rice wine etc. Spicy, raw and cold foods were strictly restricted for them. 77.9% of the participants believed that cold foods are prohibited, such as fruit, cold drinks, vegetables and cooling foods. The daily intakes of food items consumed by the participants during the puerperium are summarized in Table 2. They consumed abundant eggs, fish, poultry and meats, but the consumption of fruit, vegetables and milk were not enough. Of all the participants, 18.0% never ate vegetables, 78.8% never ate fruit and 75.7% never drank milk. Urban participants consumed significantly more fruit and milk than their rural counterparts. The consumption of sugar was excessive (mainly brown sugar), especially women in the rural area. Women residing in rural area consumed more vegetables than those who living in city.

74.6% of the women's diets were arranged by their mothers or mothers-in-law. 41.9% of the women returned to regular diets from the second month after giving birth. 55.9% of the participants (73.2% in the urban, 61.5% in the suburban and 35.6% in the rural area) had attended various kinds of nutrition courses during pregnancy.

#### Influencing factors of specific dietary behaviours

In the multiple logistic regression analysis, the response variables were milk intake (0 = no, 1 = yes), fruit intake (0 = no, 1 = yes) and vegetable intake (0 = no, 1 = yes). The results of the final models showed that living in countryside was the negative influencing factor of milk intake, whereas residing in city, attending nutrition education courses, having a higher education level and a higher family income was the positive influencing factors of milk intake. A decreased intake of fruit was correlated with living in rural area and believing that fruit was cold in nature; Living in urban area, a higher education level, a higher family income, attending nutrition education courses and having the knowledge that fruit intake is allowed while 'sitting month' increased the intake of fruit. Meal cooked by mother or mother in-law was negatively associated with vegetables intake; Residing in rural area, a higher education level of husband and having the knowledge that vegetables intake is allowed when 'sitting month' were positively associated with vegetable intake (Table 3).

### Health behaviours

#### Prevalence of health behaviours

Women in these three areas still adhered to the traditional postpartum behaviour taboos to different degrees (Table 4). 25.2% of the women believed that they should stay inside and be protected from wind, otherwise it may cause illness. These traditional concepts mainly came from mother in-law (49.4%), mother (34.0%), books and magazines (16.5%), relatives (14.3), friends or colleagues (12.6%).

Of all the participants, 54.9% didn't get out of the bed two days after giving birth. The average time they stayed in bed during the puerperium was 18.0 ± 3.8 h (16.3 ± 4.2 h in the city, 18.5 ± 3.3 h in the suburb, 19.0 ± 3.0 h in the rural). 53.7% of the women wore more clothes than usual to keep warm. 1.8% never got out of the bed except for basic needs. 32.7% never had outdoor activities. 80.0% never participated in physical exercises, only 3.8% exercised regularly (everyday or every week) during this period. 19.1% of the participants engaged in sexual intercourse within 30 days after giving birth. 18.7% initiated between 30 ~ 42 days after giving birth. 62.1% started after 42 days. The earliest sexual intercourse initiation among the participants was at the 10th day after delivery.

#### Influencing factors of specific health behaviours

In the multiple logistic regression analysis, the response variables were: getting up within two days after giving birth (0 = no, 1 = yes), doing physical exercises (0 = no, 1 = yes), complying with traditional behaviour taboos (0 = no, 1 = yes). The results showed that a higher education level of husband was the positive influencing factor for getting up within two days after giving birth, whereas living in rural area, delivering by cesarean section and having serious obstetric complication were found to be the negative influencing factors. Residing in the city, receiving a postpartum home visit by a health professional and having a higher education level were positively associated with doing physical exercises. An increased risk of complying with traditional behaviour taboos (never bathing, washing hair, brushing teeth, basking in the sunshine or ventilating the room) were found to be correlated with residing in the city and having serious obstetric complications; having a higher education level, receiving postnatal visits and attending postpartum health care education courses could decrease the risk of complying with traditional behaviour taboos (Table 5).

## Discussion

The objective of the present study was to study the postpartum practices and their influencing factors during the puerperium. In the published reports, postpartum practices were studied with a small sample of women and in one area [5,11-13]; therefore, the results might not be representative. Unlike previous studies, the design of the current study was unique, in which the participants were women who living in urban, suburban or rural areas; they represented diversified social-economical status, educational backgrounds and living situations of postpartum women.

The results of the dietary survey showed that the women consumed a variety of foods during the puerperium. This was in accordance with the contemporary nutrition principles. However, the consumption of meat, fish, poultry and eggs was excessive [14,15], especially in the rural area. The average daily consumption of egg was 365 g per person, and this was about seven times greater than the daily recommendation for Chinese [15]. Furthermore, of all the women, 18.0% never ate vegetables, 78.8% never ate fruit and 75.7% never drank milk during this period. This does not provide sufficient nutrients to meet the daily requirements of calcium, vitamin C and dietary fiber for postpartum women [14].

According to the results, the traditional behaviour taboos were still common among the participants. There was a lack of clear evidence that these behaviours are beneficial or harmful, but most of the participants complained that they were extremely uncomfortable because of not bathing, washing their hair or brushing their teeth for such a long period. Thus the life quality would be negatively influenced. More than half of the women stayed in bed for 2 days after giving birth. In the first postpartum month, they stayed in bed most of the day; nearly one third of them never had outdoor activities. These traditional postpartum behaviours would cause a series health problem like constipation [4], excessive weight gain [16] etc. Further prospective research should be carried out to explore the relationship between traditional postpartum practices and women's health outcomes.

The factors that influence the postpartum dietary and health behaviours were studied. The traditional postpartum convalescent concepts impacted women's postpartum dietary and health behaviours greatly, especially those who residing in rural area or being less educated. Higher education level was positively associated with milk and fruit intake, doing physical exercises and not complying with traditional behaviour taboos. This is in accordance with the published report [17]. Why did higher levels of education translate into not following some of the traditional postpartum practices? The assumption is that better educated women have a better understanding and resources, therefore, a better acceptance of updated health care information.

Living in rural area was negatively associated with the consumption of milk and fruit, as well as getting up within two days after giving birth. Despite the economic reforms that have been taken place in the recent decades, the economical development in rural area is lagging behind the urban, and transportation is not as convenient as that of urban area. Therefore, rural women are in a relatively closed environment as compared to their urban counterparts. They have less access to modern ideas and information. Furthermore, a lower education level is more frequent among rural women. Nevertheless, living in a rural area was positively associated with vegetable intake. This is most likely because a higher proportion of families in this area grow vegetables for self-eating, vegetables are their daily basic foods in addition to rice. Thus, strategies promoting milk and fruit intake should be targeted specifically to women with a lower level of education and to those who live in rural area.

Postpartum home visit plays an important role in helping women to change behaviours. It was positively associated with doing physical exercises and not adhering to traditional behaviour taboos. Therefore, postpartum visits should be extended to all the puerperal women.

Attendance at nutrition and health care education courses resulted in an increased consumption of fruit and a decreased tendency of complying with behaviour taboos. Health education influences one's nutrition and health care knowledge, behaviour and attitude toward postpartum practices. Thus, when promoting contemporary postpartum practices, health education courses should be encouraged.

Higher education level of husbands was positively associated with consumption of vegetables and getting up within two days after delivery. Puerperal diet arranged by mother or mother-in-law was the negative influencing factor of vegetable intake. These results suggested that husbands as well as other family members should also be involved in health education courses to ensure their support for the postpartum women and a positive attitude toward contemporary postpartum practices.

One limitation of our study was that the postpartum food intakes as well as all other data were self-reported by the women and after the completion of the puerperium, so there is potential for imprecision and recall bias. However, participants did not seem to have any difficulty in recalling the detailed dietary and health behaviours during the interview. Because of the one-child policy in China, most of our participants were primipara and 'sitting month' is recognized as a very important event in the life of a woman in China. They may have detailed memories for postpartum. In many cases, women could remember how many eggs they had eaten during the puerperium for many years. The other limitation of this study was that the participants that we had recruited were mainly Han women, whereas the postpartum practices vary among different national minorities in China. Further research on different minority populations will be very interesting.

## Conclusion

In conclusion, the present study investigated and, thereafter, provided a better understanding of the postpartum practices and the associated factors in Hubei, China. Based on our results, we recommend that the antenatal clinic should develop education programs on postpartum nutrition and health care for pregnant women and their family members. We also recommend that some of the antenatal visits be extended to early postnatal visits to follow up and guide the women on contemporary postpartum practices, which will enable women to practice them.

## Competing interests

The author(s) declare that they have no competing interests.

## Authors' contributions

Author 1(NL) participated in data collection and analysis and drafted the manuscript. Author 2(LM) had made substantial contributions on the design of the study, participated in data collection, performed the statistical analysis and helped to draft the manuscript. Author 3(XS) supervised the design and execution of the study. Authors 4(LL) participated in coordination of the study and revised the manuscript. Author 5(BC) and Author 6(QD) carried out the data collection and analysis. All authors read and approved the final manuscript.

## Pre-publication history

The pre-publication history for this paper can be accessed here:



## Figures and Tables

**Table 1:** Characters of the participants (n = 1975)

	Urban (n = 637)		Suburban (n = 628)		Rural (n = 710)		Total (n = 1975)	
	
	n	%	n	%	n	%	n	%
**Age (years)**								
< 25	157	24.7	204	32.5	216	30.4	577	29.2
25–30	364	57.1	329	52.4	291	41.0	984	49.8
> 30	116	18.2	95	15.1	203	28.6	414	21.0

**Education**								
Primary school and lower	22	3.5	30	4.8	288	40.6	340	17.2
Middle school	214	33.6	365	58.1	400	56.3	979	49.6
High school	223	35.0	165	26.3	22	3.1	410	20.8
College and above	178	27.9	68	10.8	0	0	246	12.5

**Husband's education**								
Primary school and lower	9	1.4	21	3.3	113	15.9	143	7.2
Middle school	183	28.7	360	57.3	515	72.5	1058	53.6
High school	218	34.2	166	26.4	81	11.4	465	23.5
College and above	227	35.6	81	12.9	1	0.1	309	15.7

**Occupation**								
Laborer	136	21.4	122	19.4	7	1.0	265	13.4
Farmer	33	5.2	186	29.6	689	97.0	908	46.0
Technical	62	9.8	63	10.0	3	0.4	128	6.5
Government official	57	8.7	8	1.3	0	0	65	3.3
Trader	69	10.9	18	2.9	1	0.1	88	4.5
House duty	279	43.9	231	36.8	10	1.4	520	26.3

**Parity**								
Primiparous	591	92.8	567	90.3	482	67.9	1640	83.0
Multiparous	46	7.22	61	9.71	228	32.12	335	16.96

**Delivery way**								
Cesarean section	334	52.4	239	38.1	74	10.4	647	32.8
Vaginal delivery	303	47.6	389	61.9	636	89.6	1328	67.2

**Table 2:** Average foods intakes of women in the puerperium (g/per day)

**Area**	**Cereals**	**Eggs**	**Milk**	**Meat, poultry**	**Fish, shrimp**	**Soybean**	**Vegetables**	**Leafy vegetables**	**Fruits**	**Nut**	**Sugar**
**Urban**	321.4	166.7	137.3	369.7	131.0	36.2	193.5	48.5	50.4	9.0	26.2
**Suburban**	284.2	205.7	42.5	233.1	120.4	13.6	186.2	40.3	31.0	2.4	59.5
**Rural**	387.8	365.0	4.6	256.0	67.6	46.4	424.2	222.8	7.0	0.1	81.3

**Table 3:** Factors influencing milk, fruits and vegetables intake

Response variables	Independent variables*	Estimate	Standard error	P value	Standardized Estimate	OR	95%CI
**Milk intake**	Location – rural	1.342	0.225	< 0.001	0.358	3.83	2.46~5.95
	Location – urban	-1.052	0.143	< 0.001	-0.270	0.35	0.26~0.46
	Woman's education level	-0.416	0.069	< 0.001	-0.255	0.66	0.58~0.76
	Family income	-0.303	0.072	< 0.001	-0.148	0.74	0.64~0.85
	Attending nutrition education courses	-0.467	0.155	0.003	-0.115	0.63	0.46~0.85

**Fruit intake**	Attending nutrition education courses	-0.781	0.161	< 0.001	-0.192	0.46	0.33~0.63
	Knowledge that fruits intake is allowed when 'sitting month'	-0.685	0.134	< 0.001	-0.188	0.50	0.39~0.66
	Location – rural	0.601	0.182	0.001	0.161	1.82	1.28~2.61
	Location – urban	-0.551	0.151	< 0.001	-0.143	0.58	0.43~0.78
	Family income	-0.237	0.072	0.001	-0.116	0.79	0.69~0.91
	Belief that fruit are cold in nature	0.368	0.127	0.004	0.101	1.45	1.13~1.85
	Woman's education level	-0.141	0.067	0.035	-0.087	0.87	0.76~0.99

**Vegetable intake**	Location – rural	-1.646	0.101	< 0.001	-0.443	0.19	0.16~0.24
	Knowledge that vegetable intake is allowed when 'sitting month'	-0.520	0.086	< 0.001	-0.143	0.59	0.50~0.70
	Diet arranged by mother or mother-in-law	0.313	0.093	0.001	0.077	1.37	1.14~1.64
	Husband's education level	-0.095	0.046	0.037	-0.054	0.91	0.83~0.99

*The independent variables above were selected by stepwise regression to enter into the final models. All potential independent variables were listed in the statistical analysis section.

**Table 4:** Behaviour taboos of women during the puerperium

Behaviour taboos	Urban (n = 637)		Suburban (n = 628)		Rural (n = 710)		Total (n = 1975)	
	
	n	%	n	%	n	%	n	%
Ventilate the room	82	12.9	79	12.6	37	5.2	198	10.0
Bask in the sunshine	215	33.8	164	26.1	212	29.9	591	29.9
Bath	205	32.2	164	26.1	114	16.1	483	24.5
Wash hair	216	33.9	181	28.8	241	33.9	638	32.3
Brush teeth	87	13.7	103	16.4	73	10.3	263	13.3
Feed colostrum	61	9.6	65	10.4	90	12.7	216	10.9

**Table 5:** Factors influencing health behaviours

Response variables	Independent variables	Estimate	Standard error	P value	Standardized Estimate	OR	95%CI
**Get up within 2 days after giving birth**	Husband's education level	-0.162	0.052	0.002	-0.093	0.85	0.77~0.94
	Location – rural	0.614	0.113	< 0.001	0.163	1.85	1.48~2.31
	Cesarean delivery	1.137	0.113	< 0.001	0.294	3.12	2.50~3.89
	Having serious obstetric complication	0.457	0.171	0.007	0.074	1.58	1.13~2.21

**Physical exercises**	Woman's education level	-0.332	0.054	< 0.001	-0.206	0.72	0.65~0.80
	Location – urban	-0.873	0.117	< 0.001	-0.225	0.42	0.33~0.53
	Receiving postpartum visits	-0.378	0.150	0.012	-0.076	0.69	0.51~0.92

**Behaviour taboos**	Woman's education level	0.297	0.049	< 0.001	0.183	1.35	1.22~1.48
	Location – urban	-0.273	0.114	0.017	-0.071	0.76	0.61~0.95
	Having serious obstetric complication	-0.408	0.167	0.014	-0.066	0.67	0.48~0.92
	Receiving postpartum visits	0.336	0.131	0.011	0.067	1.40	1.08~1.81
	Attending health care education courses	0.826	0.110	< 0.001	0.199	2.28	1.84~2.83

*The independent variables above were selected by stepwise regression to enter into the final models. All potential independent variables were listed in the statistical analysis section.
